# Beneficial Effects of Quercetin on Microcystin-LR Induced Tight Junction Defects

**DOI:** 10.3389/fphar.2021.733993

**Published:** 2021-09-10

**Authors:** Yuan Zhou, Mei Xue, Yunfei Jiang, Miaomiao Zhang, Changming Wang, Xuyang Wang, Guang Yu, Zongxiang Tang

**Affiliations:** ^1^ Department of Physiology, School of Medicine & Holistic Integrative Medicine, Nanjing University of Chinese Medicine, Nanjing, China; ^2^ College of Traditional Chinese Medicine·College of Intergrated Chinese and Western Medicine, Nanjing University of Chinese Medicine, Nanjing, China; ^3^ Department of Emergency, Nanjing Drum Tower Hospital, The Affiliated Hospital of Nanjing University Medical School, Nanjing, China; ^4^ Department of Neurosurgery, Shanghai Jiao Tong University Affiliated 6th People’s Hospital, Shanghai, China

**Keywords:** micorcystin-LR, quercetin, tight junction, Akt, ROS

## Abstract

Quercetin has numerous functions including antioxidant and anti-inflammatory effects. The beneficial effect of quercetin against microcystin-LR (MC-LR)-induced testicular tight junctions (TJs) defects *in vitro* and *in vivo* were investigated. Significant reductions in transepithelial electrical resistance, occludin, and zonula occludens-1(ZO-1) levels were detected in the MC-LR-treated TM4 cells, and quercetin attenuated these effects. Interestingly, quercetin suppressed MC-LR-induced phosphorylation of protein kinase B (AKT). It effectively inhibited the accumulation of reactive oxygen species (ROS) in cells stimulated by MC-LR. In addition, ROS inhibitors blocked the TJ damage that is dependent on the AKT signaling pathway induced by MC-LR. In conclusion, our results suggest that alleviates MC-LR-impaired TJs by suppressing the ROS-regulated activation of the AKT pathway.

## Introduction

Microcystins (MCs), a cyclic heptapeptide, regard as major hazards to mammals (Hu et al., 2016). It has detected more than 100 MCs variants, of which microcystin-LR (MC-LR) is the most widely studied (Zhou et al., 2014). Accumulating evidence has shown that MC-LR has various toxic effects containing hepatotoxicity and neurotoxicity (Hinojosa et al., 2019; Wang et al., 2019). In decades, laboratory experiments suggest that male gonad is a considerable target of MC-LR. The blood-testis barrier (BTB) is mainly composed of tight junctions (TJs), adherent junctions, and gap junctions (Squadrito et al., 2016). TJs which are observed between Sertoli cells play a pivot role in BTB integrity (Zhou et al., 2018). MC-LR induces TJ dysfunction, resulting in sperm abnormality (Zhang et al., 2013; Chen et al., 2018; Zhang et al., 2018).

Since the impairment of TJs induced by MC-LR is serious and irreversible, effective methods seem necessary. Several available MC-LR antidotes, such as antioxidant enzyme resultants and calcium channel blockers have been applied to reduce MC-LR toxic effects (Zhang et al., 2014; Augusti et al., 2017). Reactive oxygen species (ROS), a group of reactive, transient oxygenated substances, can regulate intracellular signal transduction pathways (Jiang et al., 2018). MC-LR-mediated Sertoli cells damage has been demonstrated to be associated with ROS production (Li and Han, 2012). Quercetin, a prominent dietary antioxidant abundantly present in vegetables, is the most active scavenger of ROS (Boots et al., 2011; Ganesan et al., 2012). It has multiple biological effects against many diseases, including renal injury and ischemic heart disease (Liu et al., 2013). Moreover, quercetin is beneficial for sperm quality and serum male hormones in diabetic rats (Abarikwu et al., 2012; Abd-Ellah et al., 2016). In addition, quercetin can prevent MC-LR-induced immune toxicity in fish lymphocytes (Zhang et al., 2014). Thus, it is hypothesized that quercetin may have a protective effect on Sertoli cells. Above all, the present study aimed to evaluate the possible beneficial effects of quercetin against MC-LR-induced TJs dysfunction.

## Materials and Methods

### Toxins

MC-LR with a purity of >96% was from Enzo Life Science (Lausen, Switzerland). MC-LR was dissolved in dimethyl sulfoxide and then kept at −20°C and diluted into media on the day of the experiment.

### Animals Experiment

The mice used in this study were male C57BL/6J mice aged from 8 to 10 weeks (Vital River Laboratory Animal company, Shanghai, China). The animals were kept in a controlled environment of 20–24°C, the humidity of 45–65%, with a 12 h day/night cycle. Forty male mice were randomly divided into four groups, each group contains 10 mice. The first group received 0.2 ml saline for 21 days. The second group was treated with 75 mg/kg quercetin for 7 days and was followed by concomitant administration of 0.2 ml saline for 14 days. In the third group, intraperitoneal injection with MC-LR at the dose of 30 μg/kg body weight was started on day 7 after the daily administration of 0.2 ml saline and was followed for 14 days. The fourth group first got 75 mg/kg quercetin for 7 days and then followed by 30 μg/kg MC-LR for 14 days. After that, testes were collected and weighted. All experiments were conducted in accordance with the protocols approved by the Animal Care and Use Committee in the Nanjing University of Chinese Medicine.

### Histology

Histopathological evaluation was conducted (Chen et al., 2011). In brief, the mice were anesthetized and perfused with 0.1 M ice-cold phosphate buffer solution (PBS) and 4% paraformaldehyde (PFA). Testis was collected and fixed in 4% PFA overnight, followed by 30% sucrose solution. The tissues were embedded in an optimum cutting temperature compound (OCT, Leica, Buffalo Grove, IL) for the frozen section. The images were observed under a microscope (Carl Zeiss, Thornwood, NY).

### Serum Hormone Assay

The blood samples (about 1.0 ml) were obtained from the mice’s eye. Blood samples were incubated at 25°C for 4 h. Then the samples were centrifuged at 3,000 rpm for 30 min serum was collected. A total of 10 mice per group were detected. Serum testosterone was measured by radioimmunoassay according to previous studies (Chen et al., 2011).

### *In vivo* BTB Integrity Assay

As described previously (Zhou et al., 2019), the BTB integrity was investigated by a biotin tracer. The right testes of mice were injected with i30 μl of EZ-Link Sulfo-NHS-LC-Biotin (Thermo Fisher, Rockford, IL) in the saline. The frozen section was made the same as previously and subsequently incubated with Alexa Fluor® 568-conjugated streptavidin (Thermo Fisher) at room temperature for 2 h. The sections were observed under a fluorescence microscope (Carl Zeiss). The localization of the biotin tracer was examined in 30 cross-sectioned seminiferous tubules and was expressed as mean ± SD (*n*= 4). The percentage over total tubes in each group.

### Measurement of Reactive Oxygen Species Formation

Dichloro-dihydrofluorescein diacetate (DCFH-DA) kit was applied to measure the concentration of ROS according to the manuscript instructions (Beyotime, Shanghai, China). Briefly, Sertoli cells were cultured in a 6-well culture plate (10^5^ cells per well). After treatment with Nac (10 mmol/L, 2 h) and exposure to MC-LR. The cells treated with different conditions were harvested and homogenized in PBS. DCFH-DA with a final concentration of 10 μM was added to the Sertoli cells, and the mixture was incubated in 37°C for 30 min. Then, the cell was washed with PBS. The percentage of ROS-positive cells were determined via an Accuri C6 plus flow cytometer (Franklin Lakes, NJ) at 485 nm excitation and 535 nm emission wavelength.

### Measuring Transepithelial Electrical Resistance

TM4 cells were plated (10^6^ cells/mL) in a transwell chamber with pore diameter 0.4 μm in a12-well plate (Corning, NY) and treated with various conditions for 24 h. The TER was detected with a Millicell ERS (Millipore, Bedford, MA) based on the previous study (Hu et al., 2016). The values were measured in each unit at three different areas and were recorded as “R”. Convert the blank corrected value to the Unit Are of Resistance by multiplying it by the effective surface area of the transwell filter insert. Resistance of a unit area = Resistance (Ω) × Effective Membrane Area (cm^2^). Four wells in each group were detected. Three independent experiments were performed.

### Western Blot

Total protein was isolated from Sertoli cells following various treatments. Homogenates of cells were prepared in lysis buffer contained with protease inhibitors cocktails (Beyotime). Protein concentration was measured by bicinchoninic acid (BCA) protein assay kit (Beyotime). 50 μg protein for each sample was separated on SDS/PAGE and electrophoretically transferred to a polyvinyoidene fluoride (PVDF) membrane (Millipore) by standard procedures. The membrane was blocked in 5% bovine serum albumin for 1 h at room temperature. MC-LR (1:500) (Enzo), zonula occludens-1 (ZO-1) (1:500), occludin (1:1,000), protein phosphatase 2A catalytic subunit (PP2Ac) (1:1,000), protein kinase B (AKT) (1:1,000), phosphorylation of AKT (p-AKT) (1:1,000), and GAPDH (1:1,000) primary antibodies and secondary anti-rabbit antibody labelled with horseradish peroxidase (HRP) were applied. The antibodies were purchased from Univ-bio (Shanghai, China). Immunoreactive bands were visualized using enhanced chemiluminescence (Tigen, Beijing, China). The figures shown represent at least three independent assays. The densitometric data were collected using Image J software (National Institutes of Health) for further statistical analysis. GAPDH was used as the loading control. Band density of interested proteins was normalized in relation to control and the results showed in percentage over relative values.

### PP2A Activity Assay

PP2A activity was determined using a serine/threonine phosphatase assay kit (Promega, Madison, WI) (Huang et al., 2017). Cells were washed with PBS and lysed on ice lysis buffer containing protease inhibitor complex for 30 min. Free phosphate was removed by Sephadex G25 column. Protein concentration was detected using the BCA method. Protein samples (2.5 μg) were evenly distributed in the 96-well plates with the PP2A-specific reaction buffer contained specific peptide substrate and incubated at 37 C for 30 min. Then 50 μl molybdate dye/additive mixture was added and allowed to incubate for 30 min. The samples were analyzed through a plate reader at an absorbance of 600 nm. The absorbance was corrected by determining that of control reactions without phosphoprotein substrate. The amount of phosphate released (pmol) was calculated from a stander curve and normalized to control. All determinations were measured in triplicate. The assay was repeated three times independently.

### Statistical Analysis

All the data are presented as mean ± SD from three independent experiments. SPSS (SPSS Inc., Chicago, IL) performed all calculations and statistical analyses. One-way analysis of variance (ANOVA) was used to analyze the difference between groups, followed by Dunnett’s *t*-test. The significance level was set at *p < 0.05*.

## Results

### Microcystin-LR Affects Testicular Tight Junctions and *p*-AKT Expression in TM4 Cells

**FIGURE 1:**
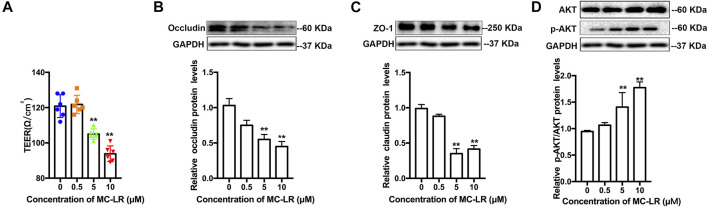
MC-LR affects TJs and *p*-AKT expression in TM4 cells. TM4 cells were cultured for 24 h, and then subjected to various concentrations of MC-LR for 24 h. **(A)** TER values were detected in TM4 cells. The protein level of occludin **(B)**, ZO-1**(C)**, AKT, and *p*-AKT **(D)** are determined through western blot (upper panels), and the protein levels were quantified with Image J (down panels). The loading control images are re-used for illustrative purposes. The results were obtained from three independent experiments. (* p < 0.05, ** p < 0.01, compared with control.)

The level of TER attenuated significantly at 5 and 10 μM. No statistically significant difference was observed in TER at 0 and 0.5 μM. Considering the TER reduction associated with TJs protein level, the occludin and ZO-1 protein expression were analyzed. The occludin and ZO-1 protein level was remarkably compromised following exposure to 5 and 10 μM MC-LR, while the protein level of p-AKT increased (Figure 1).

### Reactive Oxygen Species /AKT Pathway-dependent Testicular Tight Junctions Injury Induced by Microcystin-LR

**FIGURE 2:**
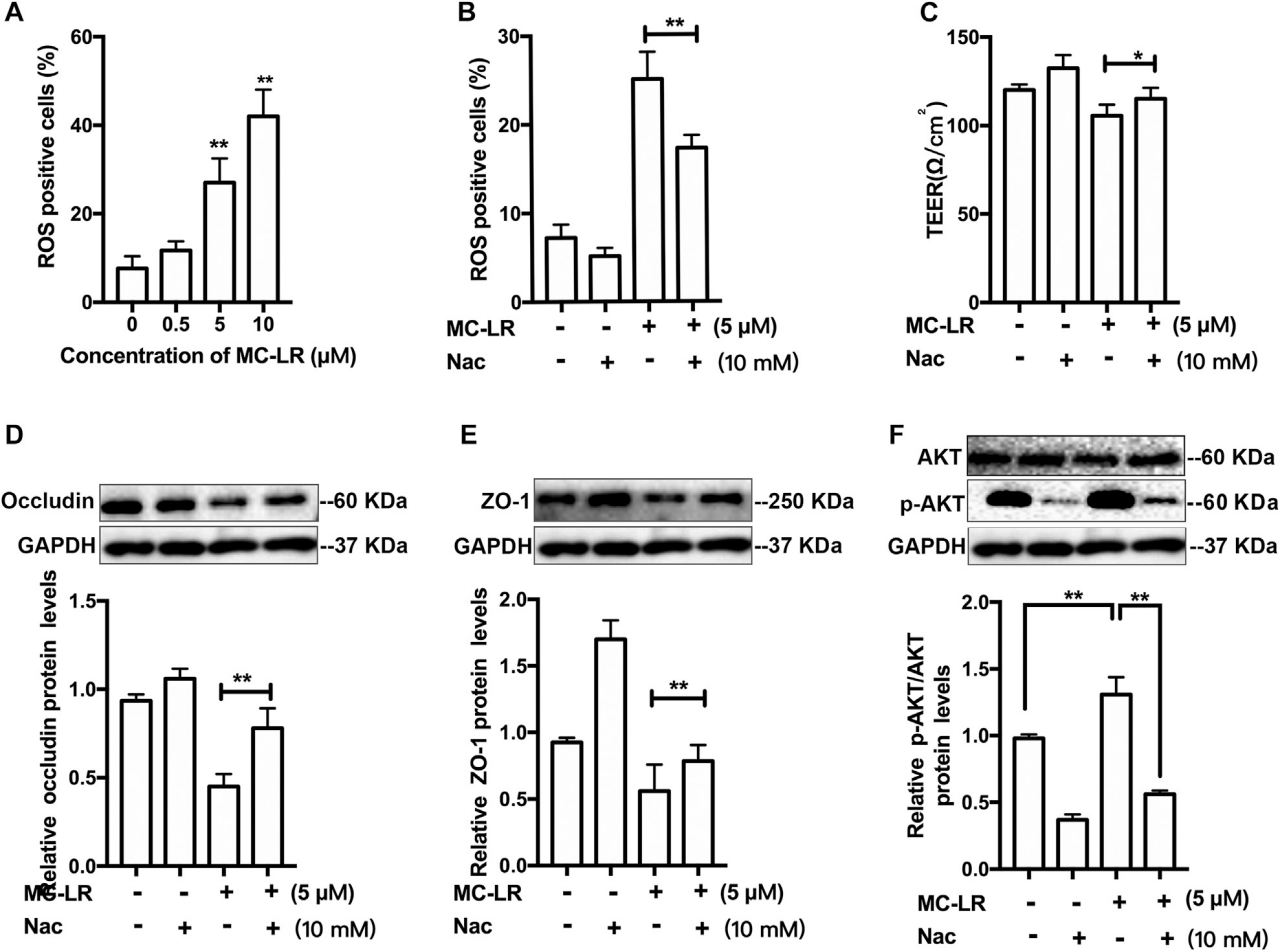
ROS/AKT pathway-dependent TJs injury induced by MC-LR. **(A)** The percentage of ROS-positive cells was measured with DCFH-DA after treatment with MC-LR. The percentage of ROS-positive cells **(B)** and the TER values **(C)** were determined with or without Nac. The proteins level of occludin **(D)**, ZO-1**(E)**, and *p*-AKT **(F)** were detected by western blot. (* p < 0.05, ** p < 0.01, compared with control.)

The concentration of ROS and phosphorylation of AKT were measured. The ROS level was elevated in a dose-dependent manner in the cells exposed to MC-LR (Figure 2A). Since a decrease in antioxidase activity was observed, ROS inhibitor Nac was further used to confirm that ROS/AKT participated in MC-LR-induced TJs injury. Nac resulted in a reduction in the percentage of ROS-positive cells, and an increase in the TER as compared with the MC-LR treatment group (Figures 2B,C). As demonstrated by the above results, MC-LR triggers the accumulation of ROS, and Nac can inhibit these effects. Pretreatment with Nac was found to rescues the MC-LR-induced downregulation of occludin (Figure 2D) and ZO-1(Figure 2E). Similarly, the p-AKT was significantly decreased with Nac pretreatment, compared with the MC-LR treatment only group (Figure 2F).

### Quercetin Alleviates Microcystin-LR-Caused Sertoli Cells Injury

**FIGURE 3:**
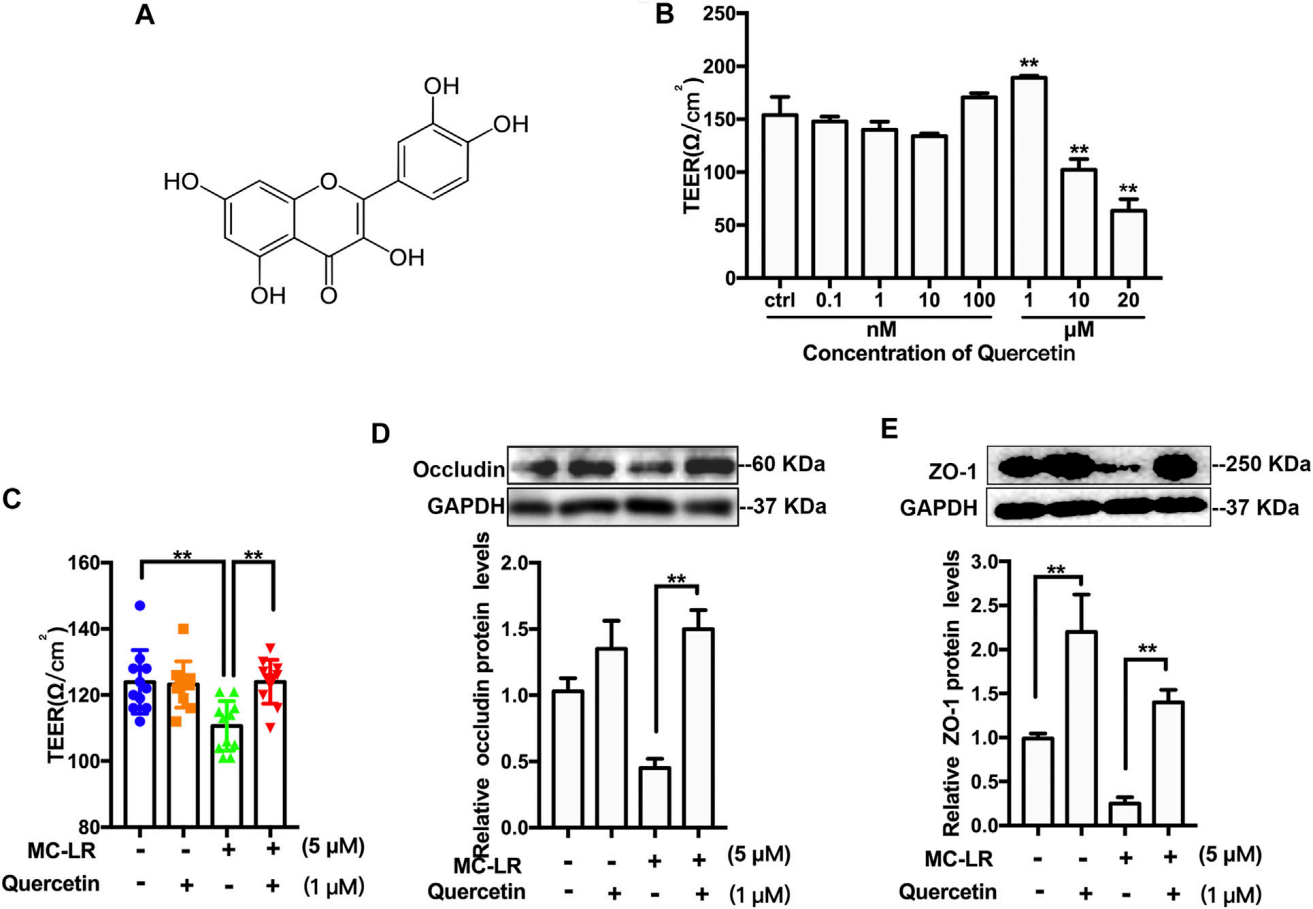
Effect of quercetin on TER and TJs protein expression. **(A)** Representative chemical structure of quercetin. **(B)** Representative the TER values of TM4 cells treated with 0.1–20 μM quercetin. **(C)** TER values of the cells exposed with quercetin (1 μM) and MC-LR (5 μM). The protein level of occludin **(D)** and ZO-1 **(E)** was measured with Western blot. Three independent experiments were performed. Each colored symbol in panel C represents one copy for three experiments after various treatments. (* p < 0.05, ** p < 0.01, compared with control.)

The structure of quercetin was present in Figure 3 A. To determine the cell membrane integrity, the TER values were detected in TM4 cells following by various treatments (Figure 3B). Quercetin treatment resulted in an obvious increase in the TER of TM4 cells at 1 μM, while a decrease at 10 and 20 μM. In addition, MC-LR remarkably attenuated the TER of TM4 cells. Treatment with quercetin, however, partially rescued the MC-LR-induced reduction in TER (Figure 3C). To further analyzed the protective effects of quercetin against MC-LR toxicity, the protein expression of occludin and ZO-1 were measured *in vitro*. MC-LR exposure downregulated the TJs protein level, whereas this decrease was reversed by quercetin administration in TM4 cells (Figures 3D,E).

### Quercetin Inhibits Reactive Oxygen Species /AKT Pathways

**FIGURE 4:**
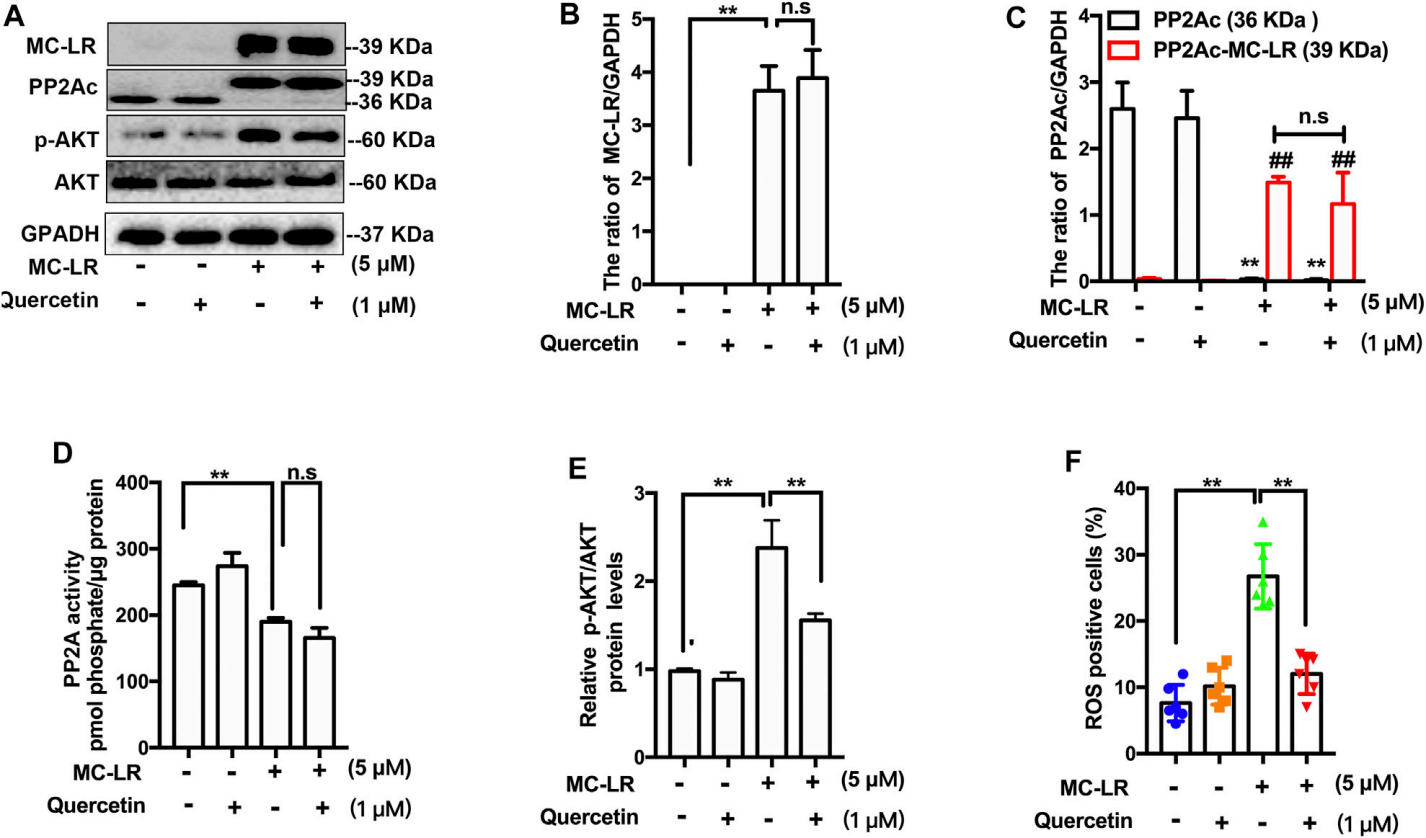
Effect of quercetin on the ROS/AKT signaling pathways. **(A)** Representative western blots showing the expression of MC-LR, PP2Ac, *p*-AKT, and GAPDH. **(B, C, E)** The expression levels were quantified with Image J. **(D)** PP2A activity was measured using commercially available assay kits. **(F)** The percentage of ROS-positive cells was determined with DCFH-DA. Three independent experiments were performed, each examined at least in triplicate. (*p < 0.05, ** p < 0.01 vs the control group; *# p < 0.05, ## p < 0.01* vs the MC-LR treatment group.).

We confirmed the association of quercetin with TM4 cells following incubation of the cells with MC-LR by western blot. However, quercetin did not affect the transportation of MC-LR into the cell (Figures 4A,B). MCs are considered to be strong inhibitors of PP2A. The expression of PP2Ac (Figures 4A,C) with MC-LR was remarkably increased and the activity of PP2A (Figure 4D) was substantially attenuated in MC-LR-exposed TM4 cells while quercetin had no significant difference in reversing these phenomena. To assess whether quercetin could inhibit MC-LR-induced TJs injury via suppressing AKT pathways, a remarkable decrease in the ratio of p-AKT/AKT was observed with quercetin treatment, compared with the MC-LR treatment only group (Figures 4A,E). To investigate whether quercetin could reduce the ROS production, TM4 cells were pre-incubated with quercetin for 2 h, and then exposed with MC-LR for 24 h. Quercetin had a similar effect on the protein level of p-AKT, which was obviously lower than that in the MC-LR treatment only group (Figure 4F).

### Quercetin Protects the Structure of Testis, Sperm Quality and Testosterone

**FIGURE 5:**
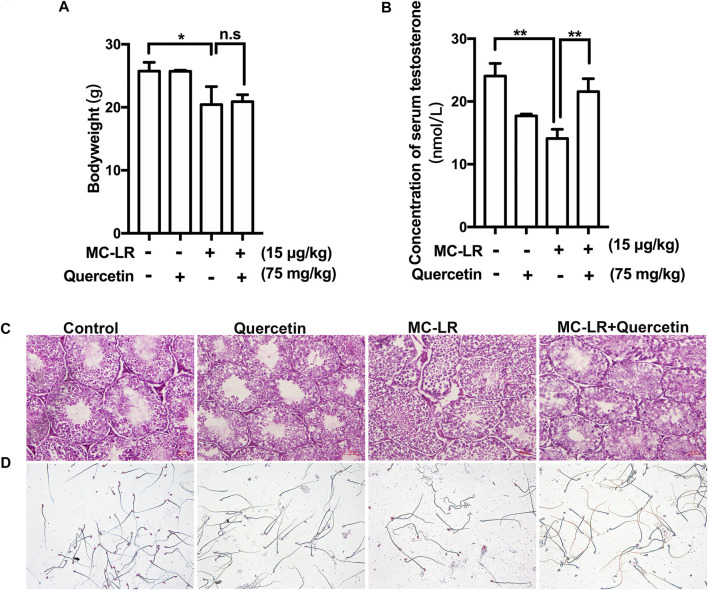
Effects of quercetin on the structure of testis, sperm quality, and testosterone. **(A)** The bodyweight. **(B)** Effect of MC-LR and quercetin on serum testosterone. **(C)** Representative illustration of histological morphology of mice testes. **(D)** Representative illustration of mice sperm. Scale bar = 50 μM. (*p < 0.05, ** p < 0.01, compared with control.)

The bodyweights of mice were not remarkably increased while the concentration of serum testosterone was upregulated in MC-LR/quercetin group compared with control in Figures 5A,B Histological staining was performed to assess the effect of quercetin on MC-LR-induced damage (Figure 5C), exhibiting that the seminiferous tubules had abnormal histological features including disorganization of the germinal epithelium and few spermatozoa in the MC-LR-treated mice compared with control. Interestingly, quercetin administration partially rescued the MC-LR-induced testicular dysfunction. The abnormality of sperm was diminished after co-treatment with MC-LR and quercetin (Figure 5D).

### Quercetin Protect the Structure of Testicular Tight Junctions

**FIGURE 6:**
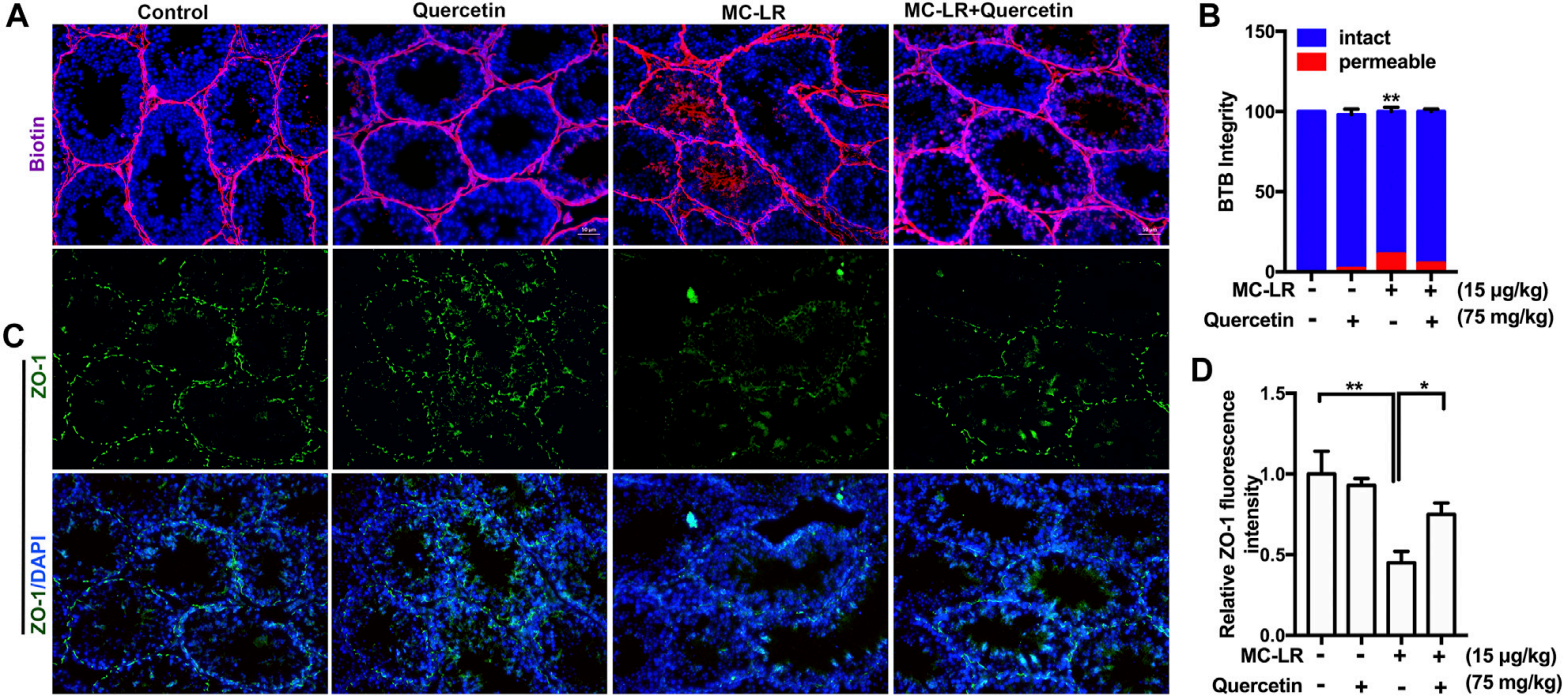
Effects of quercetin on the BTB. **(A)** Blood-testis barrier (BTB) permeability. Biotin diffusion into seminiferous tubules is marked with red fluorescence. **(B)** Percentage of seminiferous tubules with intact or permeable BTB mouse testes. The localization of the biotin tracer was examined in 30 cross-sectioned seminiferous tubules (*n* = 4). **(C)** The localization of ZO-1 (green) in the testis was examined by immunofluorescence analysis. **(D)** Quantification of the intensity of ZO-1 by image J. The cell nucleus is indicated in blue. Scale bar, 50 μM. (* p < 0.05, **p < 0.01, compared with control.)

The integrity of the BTB structure was also examined. As shown in Figures 6A,B the junctions between Sertoli cells formed tight barriers that prevent large molecules from passing through. The BTB barrier was opened after MC-LR treatment. However, Quercetin partially protected the BTB integrity from MC-LR. Similar to the *in vitro* study, quercetin rescued MC-LR-induced decrease in ZO-1 protein level (Figures 6C,D).

## Discussion

MC-LR has recently been proved to be taken up by Sertoli cells, leading to male reproductive system dysfunction (Chen et al., 2016). TJs which are the primary junctions of BTB play critical roles in an immunological barrier of host animals (Squadrito et al., 2016). MC-LR can destroy the integrity of the BTB by regulating TJs (Zhou et al., 2020). It is well known that ZO-1, claudin, and occludin are the main protein of TJs (Qiu et al., 2016), and their changes could arrest spermatogenesis, leading to the decline of sperm concentration. In our previous study, the results show that occludin and ZO-1 are significantly altered by MC-LR, while there is no effect on claudin in the *in vitro* study (Zhou et al., 2018). Therefore, ZO-1 and occludin were selected to investigate in this study. In the present study, MC-LR decreased TER values and altered the expression of TJ protein in the Sertoli cells exposure to MC-LR, which was consistent with the previous studies (Chen et al., 2018; Zhou et al., 2020). Since MC-LR has server reproductive toxicity, it is necessary to find effective drugs against the impairment of TJs induced by MC-LR.

Quercetin is a bioactive compound that is widely used in botanical medicine due to its potent antioxidant activity (Xu et al., 2019). Jahan et al. have demonstrated that quercetin has a therapeutic effect on bisphenol A-induced cytotoxicity in the male gonad (Jahan et al., 2016). Therefore, it is interesting to investigate whether and how quercetin counteracts MC-LR-caused Sertoli cell damage. To select the optimal doses of quercetin to alleviate MC-LR-induced Sertoli cells toxicity *in vitro*, TER was measured in the cells upon treatment to MC-LR from 0.1 nM to 20 μM following previous studies (Ganesan et al., 2012; Abd-Ellah et al., 2016). In our results, the TER was remarkably elevated in TM4 cells upon exposure to 1 μM quercetin. 75 mg/kg quercetin could significantly improve histological criteria and sperm parameters including sperm number, motility in NtiO_2_-intoxicated mice according to the previous researches (Khorsandi et al., 2017). Therefore, 1 μM and 75 mg/kg quercetin were chosen for *in vitro* and *in vivo* study, respectively. However, the concentrations of 10 and 20 μM quercetin decreased the TER. Thus, we cannot rule out that quercetin provides antioxidant effects at lower concentrations while inducing pro-oxidation at higher concentrations. Supplementation with quercetin inhibited MC-LR-induced TJs injury through the upregulation of ZO-1 protein level both in cells and testis. Previous studies show that MC-LR causes declines in sperm quality, decrease serum testosterone, and injury to the testis (Chen et al., 2011; Zhou et al., 2013). Treatment with quercetin could effectively reverse these phenomena.

A low level of endogenous ROS is required for the regulation of vital sperm functions (Agarwal et al., 2014). In the present study, large amounts of ROS were produced in TM4 cells exposed to MC-LR. Pretreatment with Nac (ROS-specific scavenger) could effectively inhibited the generation of ROS, increase TER value, and notably promoted the expression of TJs proteins. AKT plays an important role in anoikis resistance through its phosphorylation. Zhou et al. indicate that AKT has a commanding role in the regulation of TJs dynamics in Sertoli cells (Zhou et al., 2018). In our previous study, the AKT signaling pathway regulated the effects of MC-LR on TJ proteins, as inhibition of p-AKT could obviously rescue the MC-LR-induced downregulation of TJ protein (Zhou et al., 2018). Sufficient evidence indicates that ROS mediate intracellular signal transduction pathways (Jiang et al., 2018). Accordingly, LPS causes cell malfunction by mediating ROS-mediated PI3K/AKT (Jiang et al., 2018). Additionally, Nac attenuated the protein level of p-AKT under the MC-LR treatment condition. Taken together, these results indicate that ROS was an upstream factor of p-AKT in male Sertoli cells exposed to MC-LR.

Quercetin can remove ROS, which can resist oxidative damage induced by radiation and sperm change associated with ROS (Xu et al., 2019). Ganesan et al. have proven that pretreatment of cells with quercetin decreased with AKT phosphorylation in human airway epithelial cells (Ganesan et al., 2012). In addition, LY294002 is a potent inhibitor of AKT based on the structure design of quercetin (Zhang et al., 2014). Our results found that quercetin obviously suppressed the accumulation of ROS and reduced the expression of the phosphorylation AKT. MCs are regard as activators of oxidative stress and potent inhibitors of cellular PP2A (Meng et al., 2019). MC-LR inhibits PP2Aactivity, resulting in AKT signaling pathways activation in Sertoli cells (Zhou et al., 2018). However, the results indicated that PP2A activity was not significantly reversed by quercetin in MC-LR-treated TM4 cells.

In summary, we have shown that quercetin can protect MC-LR-induced Sertoli cell toxicity by restoring the function of TJs. Taken together, treatment with antioxidants, such as quercetin, could help maintain the high male reproductive capacity by protecting Sertoli cells from MC-LR-induced injury.

## Data Availability

The original contributions presented in the study are included in the article/supplementary material, further inquiries can be directed to the corresponding authors.

## References

[B1] Abarikwu S. O. Pant A. B. Farombi E. O. (2012). The Protective Effects of Quercetin on the Cytotoxicity of Atrazine on Rat Sertoli-Germ Cell Co-culture. Int. J. Androl. 35, 590–600. 10.1111/j.1365-2605.2011.01239.x 22372587

[B2] Abd-Ellah M. F. Aly H. A. Mokhlis H. A. Abdel-Aziz A. H. (2016). Quercetin Attenuates Di-(2-ethylhexyl) Phthalate-Induced Testicular Toxicity in Adult Rats. Hum. Exp. Toxicol. 35, 232–243. 10.1177/0960327115580602 25882133

[B3] Agarwal A. Virk G. Ong C. Du Plessis S. S. (2014). Effect of Oxidative Stress on Male Reproduction. World J. Mens Health 32, 1–17. 10.5534/wjmh.2014.32.1.1 24872947 PMC4026229

[B4] Augusti P. R. Brasil A. V. S. Souto C. Göethel G. de Oliveira Rios A. Emanuelli T. (2017). Microcystin-LR Exposure Induces Oxidative Damage in *Caenorhabditis elegans*: Protective Effect of Lutein Extracted from Marigold Flowers. Food Chem. Toxicol. 109, 60–67. 10.1016/j.fct.2017.08.045 28866331

[B5] Boots A. W. Drent M. De Boer V. C. Bast A. Haenen G. R. (2011). Quercetin Reduces Markers of Oxidative Stress and Inflammation in Sarcoidosis. Clin. Nutr. 30, 506–512. 10.1016/j.clnu.2011.01.010 21324570

[B6] Chen Y. Xu J. Li Y. Han X. (2011). Decline of Sperm Quality and Testicular Function in Male Mice during Chronic Low-Dose Exposure to Microcystin-LR. Reprod. Toxicol. 31, 551–557. 10.1016/j.reprotox.2011.02.006 21338672

[B7] Chen L. Chen J. Zhang X. Xie P. (2016). A Review of Reproductive Toxicity of Microcystins. J. Hazard. Mater. 301, 381–399. 10.1016/j.jhazmat.2015.08.041 26521084

[B8] Chen Y. Wang J. Pan C. Li D. Han X. (2018). Microcystin-leucine-arginine Causes Blood-Testis Barrier Disruption and Degradation of Occludin Mediated by Matrix Metalloproteinase-8. Cell Mol. Life Sci. 75, 1117–1132. 10.1007/s00018-017-2687-6 29071384 PMC11105681

[B9] Ganesan S. Faris A. N. Comstock A. T. Wang Q. Nanua S. Hershenson M. B. (2012). Quercetin Inhibits Rhinovirus Replication *In Vitro* and *In Vivo* . Antivir. Res. 94, 258–271. 10.1016/j.antiviral.2012.03.005 22465313 PMC3360794

[B10] Hinojosa M. G. Prieto A. I. Gutiérrez-Praena D. Moreno F. J. Cameán A. M. Jos A. (2019). Neurotoxic Assessment of Microcystin-LR, Cylindrospermopsin and Their Combination on the Human Neuroblastoma SH-SY5Y Cell Line. Chemosphere 224, 751–764. 10.1016/j.chemosphere.2019.02.173 30851527

[B11] Hu Y. Chen J. Fan H. Xie P. He J. (2016). A Review of Neurotoxicity of Microcystins. Environ. Sci. Pollut. Res. Int. 23, 7211–7219. 10.1007/s11356-016-6073-y 26857003

[B12] Huang P. Wang B. Wang X. Xing M. Guo Z. Xu L. (2017). HEK293 Cells Exposed to Microcystin-LR Show Reduced Protein Phosphatase 2A Activity and More Stable Cytoskeletal Structure when Overexpressing α4 Protein. Environ. Toxicol. 32, 255–264. 10.1002/tox.22230 26784437

[B13] Jahan S. Ain Q. U. Ullah H. (2016). Therapeutic Effects of Quercetin against Bisphenol A Induced Testicular Damage in Male Sprague Dawley Rats. Syst. Biol. Reprod. Med. 62, 114–124. 10.3109/19396368.2015.1115139 26787223

[B14] Jiang K. Guo S. Yang C. Yang J. Chen Y. Shaukat A. (2018). Barbaloin Protects against Lipopolysaccharide (LPS)-induced Acute Lung Injury by Inhibiting the ROS-Mediated PI3K/AKT/NF-κB Pathway. Int. Immunopharmacol. 64, 140–150. 10.1016/j.intimp.2018.08.023 30173054

[B15] Khorsandi L. Orazizadeh M. Moradi-Gharibvand N. Hemadi M. Mansouri E. (2017). Beneficial Effects of Quercetin on Titanium Dioxide Nanoparticles Induced Spermatogenesis Defects in Mice. Environ. Sci. Pollut. Res. Int. 24, 5595–5606. 10.1007/s11356-016-8325-2 28035607

[B16] Li Y. Han X. (2012). Microcystin-LR Causes Cytotoxicity Effects in Rat Testicular Sertoli Cells. Environ. Toxicol. Pharmacol. 33, 318–326. 10.1016/j.etap.2011.12.015 22301162

[B17] Liu C. M. Zheng G. H. Ming Q. L. Sun J. M. Cheng C. (2013). Protective Effect of Quercetin on lead-induced Oxidative Stress and Endoplasmic Reticulum Stress in Rat Liver via the IRE1/JNK and PI3K/Akt Pathway. Free Radic. Res. 47, 192–201. 10.3109/10715762.2012.760198 23249147

[B18] Meng X. Peng H. Ding Y. Zhang L. Yang J. Han X. (2019). A Transcriptomic Regulatory Network Among miRNAs, piRNAs, circRNAs, lncRNAs and mRNAs Regulates Microcystin-Leucine Arginine (MC-LR)-induced Male Reproductive Toxicity. Sci. Total Environ. 667, 563–577. 10.1016/j.scitotenv.2019.02.393 30833255

[B19] Qiu L. Qian Y. Liu Z. Wang C. Qu J. Wang X. (2016). Perfluorooctane Sulfonate (PFOS) Disrupts Blood-Testis Barrier by Down-Regulating junction Proteins via P38 MAPK/ATF2/MMP9 Signaling Pathway. Toxicology 373, 1–12. 10.1016/j.tox.2016.11.003 27818224

[B20] Squadrito F. Micali A. Rinaldi M. Irrera N. Marini H. Puzzolo D. (2016). Polydeoxyribonucleotide, an Adenosine-A2a Receptor Agonist, Preserves Blood Testis Barrier from Cadmium-Induced Injury. Front. Pharmacol. 7, 537. 10.3389/fphar.2016.00537 28119612 PMC5222826

[B21] Wang Q. Liu Y. Guo J. Lin S. Wang Y. Yin T. (2019). Microcystin-LR Induces Angiodysplasia and Vascular Dysfunction through Promoting Cell Apoptosis by the Mitochondrial Signaling Pathway. Chemosphere 218, 438–448. 10.1016/j.chemosphere.2018.11.019 30485828

[B22] Xu D. Hu M. J. Wang Y. Q. Cui Y. L. (2019). Antioxidant Activities of Quercetin and its Complexes for Medicinal Application. Molecules 24, 1123. 10.3390/molecules24061123 PMC6470739 30901869

[B23] Zhang H. Cai C. Wu Y. Shao D. Ye B. Zhang Y. (2013). Mitochondrial and Endoplasmic Reticulum Pathways Involved in Microcystin-LR-Induced Apoptosis of the Testes of Male Frog (*Rana nigromaculata*) *In Vivo* . J. Hazard. Mater. 252-253, 382–389. 10.1016/j.jhazmat.2013.03.017 23548922

[B24] Zhang H. Wu Y. Fang W. Wang D. (2014). Regulatory Effect of Quercetin on Hazardous Microcystin-LR-Induced Apoptosis of *Carassius auratus* Lymphocytes *In Vitro* . Fish. Shellfish Immunol. 37, 278–285. 10.1016/j.fsi.2014.02.015 24594009

[B25] Zhang L. Meng X. Xiang Z. Li D. Han X. (2018). From the Cover: Roles of mmu_piR_003399 in Microcystin-Leucine Arginine-Induced Reproductive Toxicity in the Spermatogonial Cells and Testis. Toxicol. Sci. 161, 159–170. 10.1093/toxsci/kfx209 29040791

[B26] Zhou Y. Chen Y. Yuan M. Xiang Z. Han X. (2013). *In Vivo* study on the Effects of Microcystin-LR on the Apoptosis, Proliferation and Differentiation of Rat Testicular Spermatogenic Cells of Male Rats Injected i.P. With Toxins. J. Toxicol. Sci. 38, 661–670. 10.2131/jts.38.661 24025782

[B27] Zhou Y. Xiang Z. Li D. Han X. (2014). Regulation of Microcystin-LR-Induced Toxicity in Mouse Spermatogonia by miR-96. Environ. Sci. Technol. 48, 6383–6390. 10.1021/es500152m 24803159

[B28] Zhou Y. Geng X. Chen Y. Shi H. Yang Y. Zhu C. (2018). Essential Roles of Akt/Snail Pathway in Microcystin-LR-Induced Tight junction Toxicity in Sertoli Cell. Food Chem. Toxicol. 112, 290–298. 10.1016/j.fct.2018.01.004 29307602

[B29] Zhou Y. Chen Y. Hu X. Guo J. Shi H. Yu G. (2019). Icariin Attenuate Microcystin-LR-Induced gap junction Injury in Sertoli Cells through Suppression of Akt Pathways. Environ. Pollut. 251, 328–337. 10.1016/j.envpol.2019.04.114 31091496

[B30] Zhou Y. Sun M. Tang Y. Chen Y. Zhu C. Yang Y. (2020). Responses of the Proteome in Testis of Mice Exposed Chronically to Environmentally Relevant Concentrations of Microcystin-LR. Ecotoxicol. Environ. Saf. 187, 109824. 10.1016/j.ecoenv.2019.109824 31654863

